# Fraction collection of bioactive compounds from ion chromatography: No longer mission impossible

**DOI:** 10.1016/j.mex.2025.103243

**Published:** 2025-02-21

**Authors:** Hans S.A. Yates, James F. Carter, Mary T. Fletcher, Viviene S. Santiago, Ondrea Thompson, Natasha L. Hungerford

**Affiliations:** a Queensland Health Coronial and Public Health Sciences (QHCPHS) Health and Food Sciences Precinct, Coopers Plains, QLD, Australia; b Queensland Alliance for Agriculture and Food Innovation (QAAFI), The University of Queensland. Health and Food Sciences Precinct, Coopers Plains, QLD, Australia; c Thermo Fisher Scientific, 76 Depot St, Banyo, QLD 4014, Australia; d School of Chemistry and Molecular Biosciences, The University of Queensland, St Lucia, QLD 4072, Australia

**Keywords:** Saccharide analysis, Sugar preparation, Honey, Trehalulose, High performance anion exchange chromatography, HPAEC, Pulsed amperometric detection, PAD, Nuclear magnetic resonance, NMR, Fraction collection of bioactive compounds by IC

## Abstract

This paper demonstrates the fraction collection of the novel sugar trehalulose, using a modified ion chromatograph. The Ion Chromatography (IC) method, previously published for the analysis of trehalulose, was augmented with a suppressor and purpose-made switching valve unit. A sample of stingless bee honey was then run, following the three main steps:

• Separation of trehalulose fraction
• Lyophilization
• Confirmation of trehalulose

The method should be applicable to not only sugar analysis but to any bioactive compound separable by IC. The authors were not able to find a similar method within currently published literature.

## Graphical abstract

**Figure:**
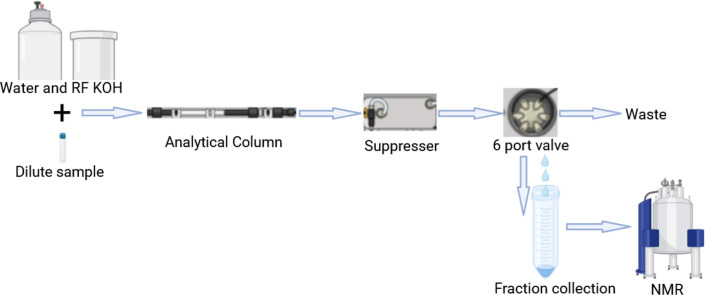

Specifications table

**Table:** 

Subject area:	Chemistry
More specific subject area:	Analytical chemistry, food chemistry, biodiscovery
Name of your method:	Fraction collection of bioactive compounds by IC
Name and reference of original method:	Hungerford, N. L., Zhang, J., Smith, T. J., Yates, H. S. A., Chowdhury, S. A., Carter, J. F., Carpinelli de Jesus, M., & Fletcher, M. T. (2021). Feeding sugars to stingless bees: identifying the origin of trehalulose-rich honey composition. *Journal of Agricultural and Food Chemistry, 69*(35), 10292-10300. https://doi.org/10.1021/acs.jafc.1c02859
Resource availability:	^1^H NMR spectrum of isolated trehalulose and NMR spectra comparative table in supplementary material

## Background

Ion Chromatography (IC) and High-Performance Liquid Chromatography (HPLC) are often compared as analytical techniques, as they both use liquid mobile phases. Unlike HPLC, IC employs highly caustic mobile phases (≈ pH 12) such as aqueous potassium hydroxide, making conventional fraction collection difficult. For saccharide analysis, IC is quoted as having better selectivity than HPLC [1]. However, in some articles, more compounds (assumed to be saccharides) were observed than could be identified by retention time, using the corresponding standards [2,1]. Therefore, a successful fraction collection method would be ideal for identification of these unknown compounds.

Ion chromatography can be coupled directly to pulsed amperometric detection (IC/PAD) but, when coupling an IC with mass spectrometery (MS), the eluent must pass through a suppressor. It is this suppressor that also makes fraction collection possible. The suppressor uses electrolysis to create a source of hydronium ions (H_3_O^+^), which are added to the caustic eluent via an ion exchange membrane. At the same time, this membrane removes cations, such as potassium (K^+^) from potassium hydroxide, from the eluent. The hydronium ions (H_3_O^+^) react with hydroxide (OH^-^) in the mobile phase to create water. Once the eluent has been neutralized, any anions still present are removed by another ion exchange membrane. Overall, the eluent is converted from strong hydroxide (MS incompatible) to water (MS compatible) [3]. In the presence of the basic mobile phase, sugars exist in their weak acid anionic forms, which make the sugars amenable to separation by ion chromatography. Within the suppressor, these sugar anions are converted back to their uncharged sugar forms. This combination of water and sugar can then pass through a diverter valve to allow collection of analytes of interest.

Once isolated, further work is needed to characterise the analyte of interest, which requires at least milligram amounts of the compounds. There is currently a lack of preparative-scale columns for IC and some compounds may only be present in minute quantities. The fractionate, therefore, needs to be collected repeatedly from multiple injections of the same sample. This creates large volumes of fractionate requiring concentration before identification or confirmation. As the fraction is primarily water, this can easily be removed by lyophilization (freeze-drying) and the target analyte is left for confirmation.

The operation of the system was demonstrated by isolation of trehalulose from a sample of stingless bee honey.

## Materials and methods

### Chemicals and honey samples

Ultra-pure water (<18.2 MΩ) was prepared onsite using a Milli-Q water purification system. Stingless bee honey samples were obtained from a specialty retailer outlet. Trehalulose (95 % purity) was purchased from Biosynth (catalogue no. OT09774), and deuterium oxide (D_2_O, 99.9 % deuterium) NMR solvent was purchased from Sigma (catalogue no. 151882).

### Apparatus

The method was developed with a ThermoFisher Dionex ICS-6000 Ion Chromatograph with a 2 mm “reagent-free” channel (ThermoFisher catalogue no. 22181–60009), AS-AP 100 µL Syringe (ThermoFisher catalogue no. 074305), AS-AP autosampler (ThermoFisher catalogue no. 074926), Automation Manager (ThermoFisher catalogue no. 079833), Dionex KOH EGC 500 cartridge (ThermoFisher catalogue no. 075778), AXP pump (ThermoFisher catalogue no. 063973), Dionex 2 mm CR-ATC 600 (ThermoFisher catalogue no. 088662), and Dionex 2 mm ADRS 600 Anion dynamic regenerated suppressor (ThermoFisher catalogue no. 088667). Chromeleon software (ThermoFisher7200.0201) was used to control the IC-MS and for post-processing of results.

### Modified automation manager and diverter valves

**Fig. 1:**
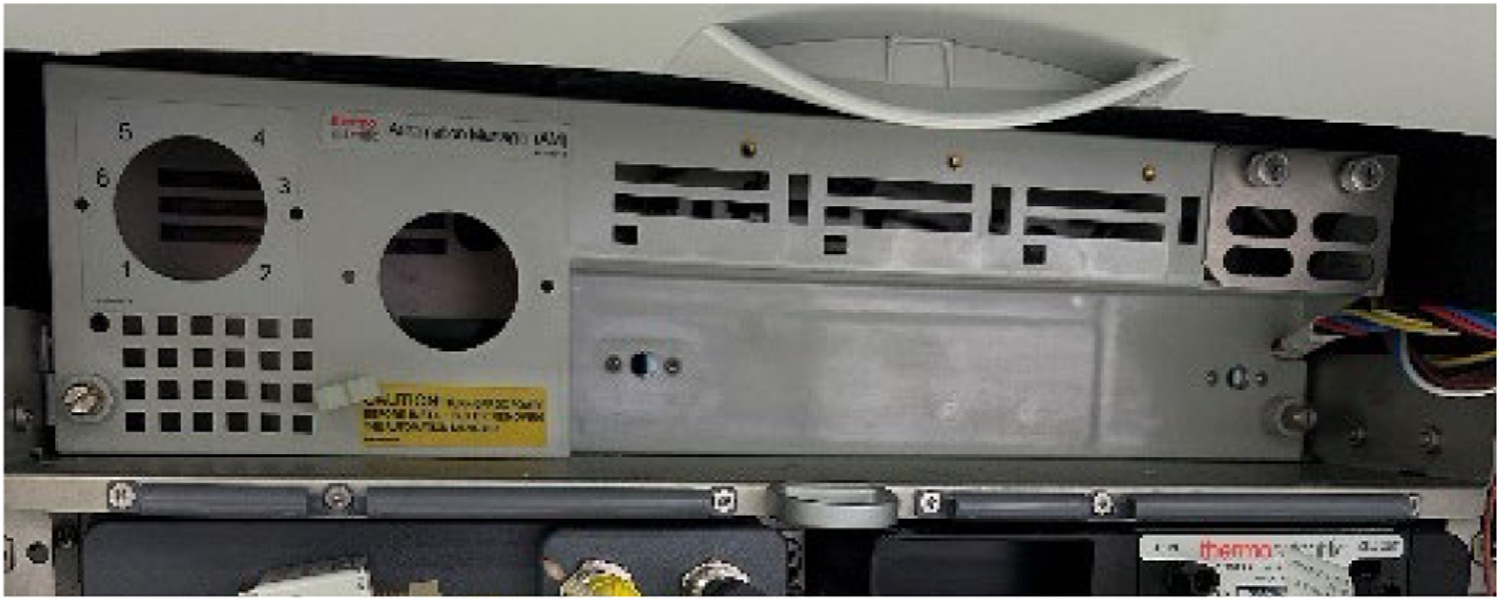
Automation manager within the IC. The round spaces where the diverter valves would normally be installed are present on the left-hand side.

**Fig. 2:**
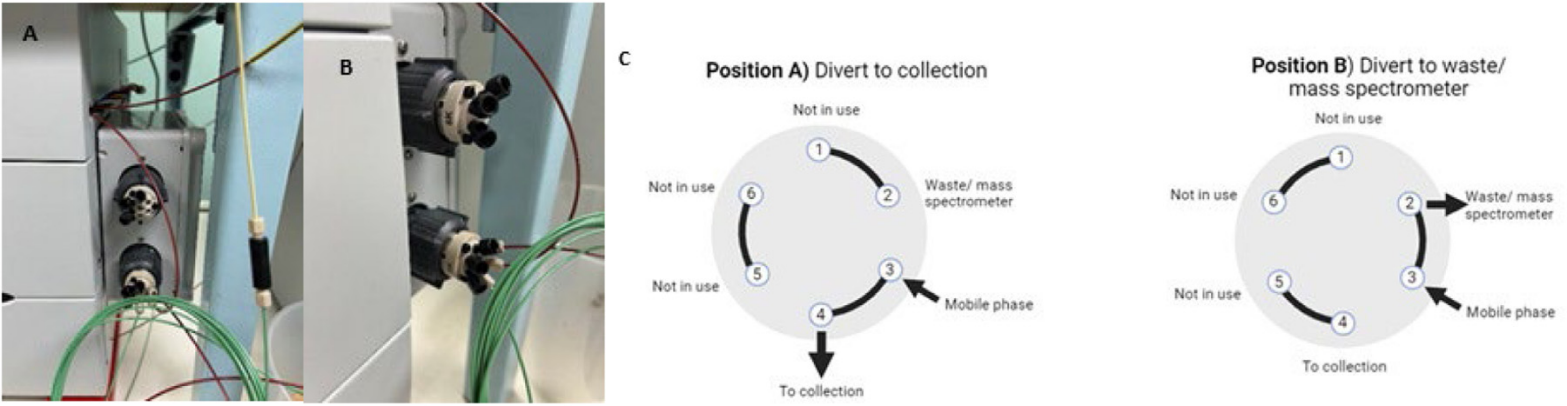
Control enclosure with diverter valves A) front on and B) side on C) schematic of valve positions A and B (Created in https://BioRender.com).

Automation manager (ThermoFisher catalogue no. 075952) is a commercially available plug-in control board for the operation of additional IC devices. In this instance, the automation manager (Fig. 1) along with two 6-port valves (ThermoFisher catalogue no. 075952) were modified so that the valves could be placed outside the IC, as per Fig. 2A & Fig. 2B. One of these valves allowed the flow of mobile phase to be diverted to either an A or B outwards configuration, as per Fig. 2C below.

**Fig. 3:**
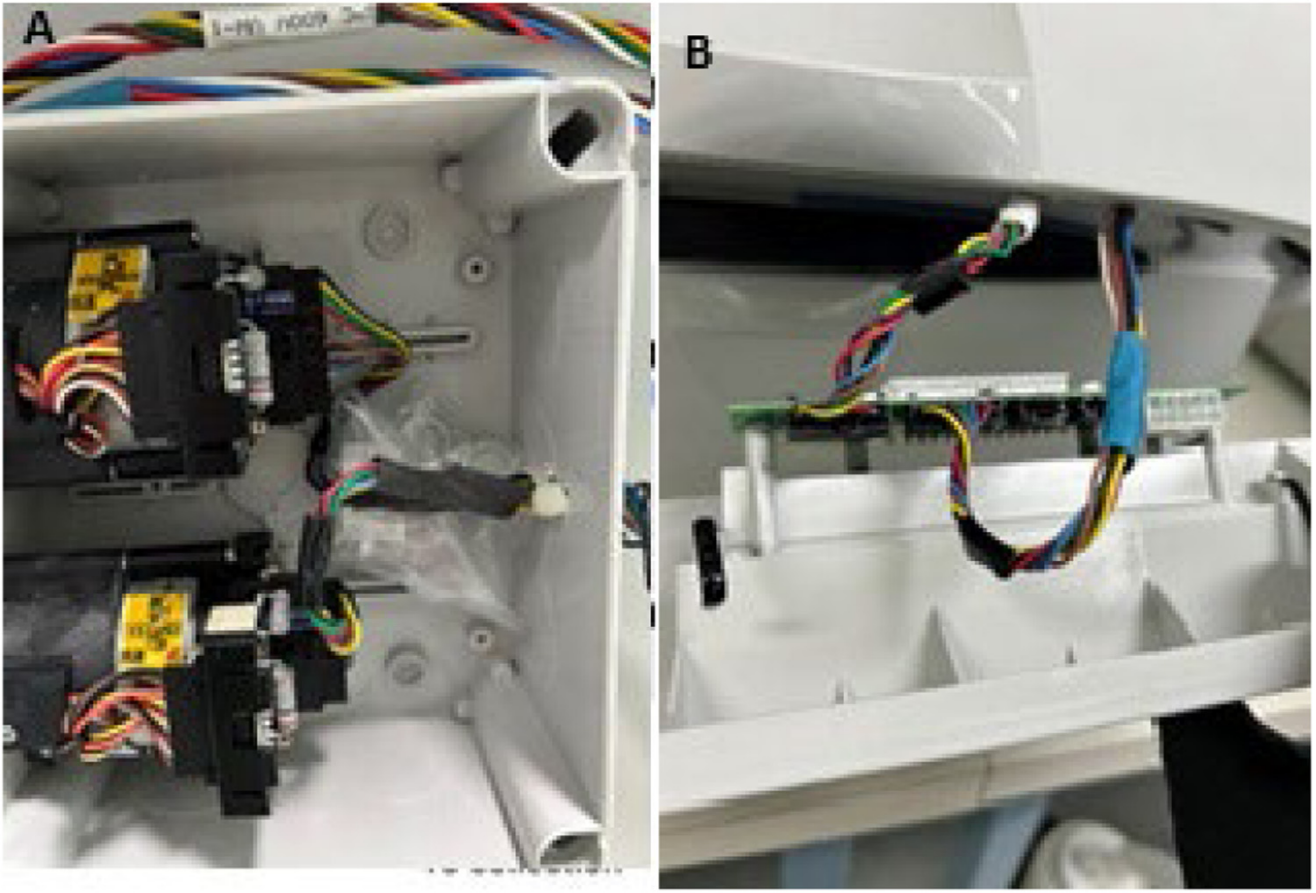
The extended cable connecting A) valve boards and B) automation manager.

This modification, performed by a qualified technician/electrician, was completed by machining a generic plastic electrical control enclosure (Lawrence & Hanson) to accommodate the valves and extend the original connection cables utilising a 6-wire electrical cable (Jaycar or RS Components). This linked the connection points on the valves (Fig. 3A) with those on the automation manager (Fig. 3B). The automation manager was configured in the instrument software (Chromeleon) as per manufacturer's instructions. The method script was updated to cover the operation of the valves. Stick-on magnets (ThermoFisher catalogue no. 22153–40107) were attached to the side of the enclosure so that it could easily be moved or repositioned as required on the side of the IC.

**Fig. 4:**
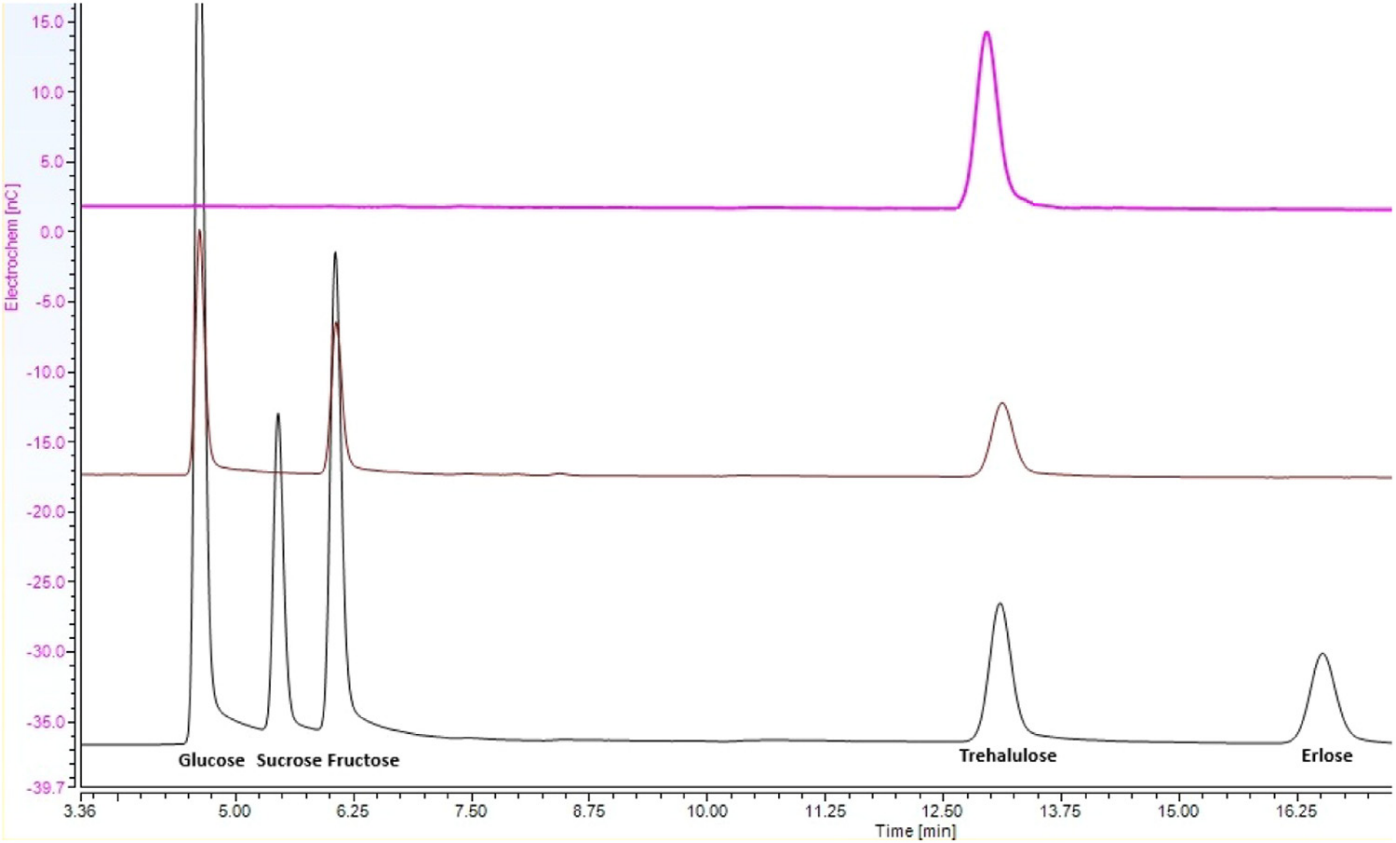
Combined IC/PAD chromatograph of trehalulose in fractionate (top, pink chromatogram), unprocessed stingless bee honey (middle, brown chromatogram) and combined mixed standards [6] containing glucose, sucrose, fructose, trehalulose and erlose (bottom, black chromatogram).

**Fig. 5:**
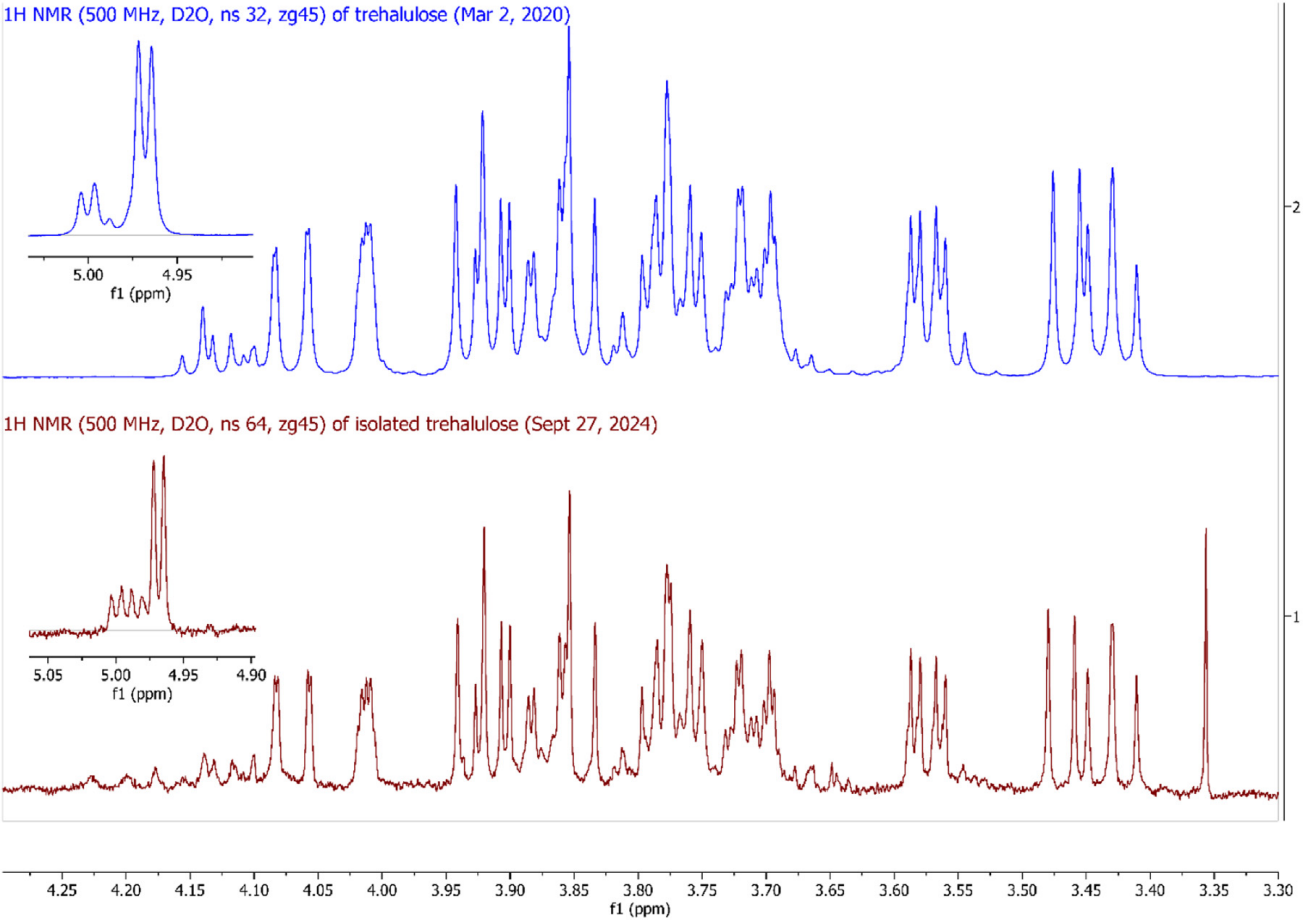
Comparison of ^1^H NMR (500 MHz, D_2_O) spectra of trehalulose from literature (top, from [4]) and isolated trehalulose in this study (bottom).

### Stingless bee honey sample preparation

A honey sample (0.3 g) was weighed into a 50 mL centrifuge tube and diluted with 30 mL of ultra-pure water (<18.2 MΩ). The tube was mechanically shaken (Heathrow Scientific catalogue no. HS120240) until all the honey was dissolved. The mixture was then centrifuged at 10,000 g, 4 °C for 10 min. The resulting supernatant was obtained and further diluted in a 1:10 ratio with ultra-pure water before transferring to amber sample vials.

### IC analysis parameters

Chromatographic separation was completed on a ThermoFisher Dionex ICS-6000 Ion Chromatograph (details above). The sample solution (4 µL) was cooled to 16 °C prior to injection onto a 2 mm CarboPac PA210 column (30 × 2 mm, 4 um, ThermoFisher catalogue no. 088956) fitted with a 2 mm CarboPac PA210 guard column (30 × 2 mm, 4 um, ThermoFisher catalogue no. 088956). The mobile phase used was a mixture of ultra-pure water and autogenerated potassium hydroxide (KOH) (Table 1). A ThermoFisher Dionex ADRS suppressor, operating at 20 °C and 40 mA, was used to neutralize the mobile phase. Neutralization occurred using the external water source mode of the AXP pump at the rate of 0.25 mL/min.

**Table 1:** IC gradient program for collection of fraction with retention time of 15 min.

Time (min)	KOH Concentration (mM)	Diverter valve (position)	Program
0	12	B	Analysis
12.5	12	A	Divert to collection
18	12	A	Divert from collection
34	12	B	
34.1	100	B	Cleaning
59	100	B	
59.1	12	B	Equilibration
79	12	B	

The honey sample was profiled first to determine the retention time of the target analyte. Using the preliminary chromatograph, a fraction collection method for ion chromatography was optimized (Table 1).

### Collection methodology

A length of red PEEK tubing (0.005 inch internal diameter) connected the valve and a centrifuge tube within the block of the Thermomixer (Eppendorf catalogue no. 5382000066 and 5365000028). The block was set to 1 °C, which resulted in an average temperature across the block of 5 °C. A calibrated data logger was present in the block for the duration of the collection.

Each injection of the honey sample resulted in approximately 3 mL of fractionate with an estimated concentration of 8 mg/L trehalulose. When the tube was approximately 40 mL full of fractionate (≈ 13 injections), it was manually swapped with an empty tube and frozen. Three 50 mL centrifuge tubes of fractionate were collected.

### Freeze drier methodology

The three frozen 50 mL tubes were lyophilized (Christ Alpha 1–4 LSC with Lyocube 4–8) for 18 h at 0.5 mbar. The resulting dried tubes were washed with small aliquots of pure water and combined in a 15 mL centrifuge tube. The 15 mL tube with sample was frozen and lyophilized using the same program yielding approximately two crystals of sugar (<1 mg).

### Nuclear magnetic resonance (NMR) methodology

A sample for ^1^H NMR analysis was prepared by dissolving the isolated solid in 500 µL of D_2_O (Sigma catalogue no. 151882) and transferring the resulting solution to a 5 mm diameter NMR tube (Sigma catalogue no. Z565229). The ^1^H NMR spectrum was acquired at ambient temperature and pressure on a Bruker Avance 500 MHz spectrometer. A zg45 pulse sequence was used with 64 scans and a spectral width of 6.5 ppm was applied. Chemical shifts were expressed in ppm and the NMR spectrum was referenced to residual protonated solvent signal (δ 4.79 ppm) using Mnova software (Mestrelab Research).

## Method validation

The presence of trehalulose in the collected fraction was confirmed by comparing the IC/PAD chromatogram of the isolated compound (Fig. 4, pink chromatogram) with a standard solution containing a mixture of glucose, sucrose, fructose, trehalulose, and erlose (Fig. 4, black chromatogram). To prove further the identity of the isolated compound, the ^1^H NMR spectrum of the isolated compound (Fig. 5, red trace) was compared to the ^1^H NMR spectrum of a trehalulose standard (Fig. 5, blue trace).

**Fig. 6:**
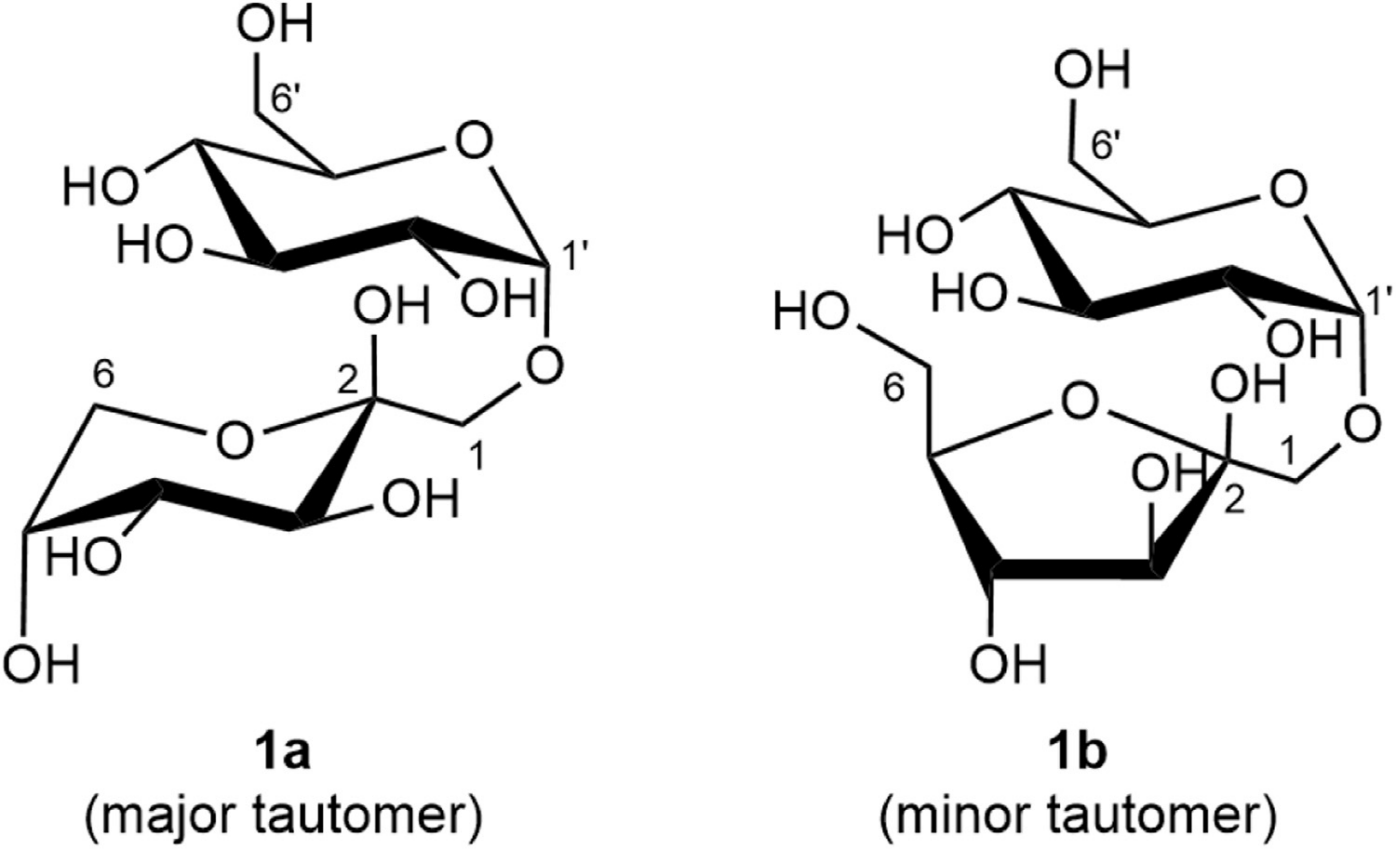
Trehalulose is isolated as a mixture of tautomers, with 1-*O*-α-d-glucopyranosyl-β-d-fructopyranose (**1a**) as the major tautomer and 1-*O*-α-d-glucosylpyranosyl-β-d-fructofuranose (**1b**) as the minor tautomer.

Comparison of the ^1^H NMR spectrum with published ^1^H NMR data from [4] (Fig. 5) confirmed that the isolated solid using the IC/PAD method was trehalulose. Trehalulose is composed of a glucose and fructose unit joined by an α-(1 → 1) glycosidic bond, with the presence of the fructose unit resulting in trehalulose existing as a mixture of tautomers in solution (Fig. 6). Since trehalulose is reported to be one of the few oligosaccharides in which the fructose ring predominantly exists in the pyranose form in solution, 1-*O*-α-d-glucopyranosyl-β-d-fructopyranose (**1a**) is observed to be the major tautomer while 1-*O*-α-d-glucosylpyranosyl-β-d-fructofuranose (**1b**) is the minor tautomer [5]. Both tautomers were detected in the ^1^H NMR spectrum of the isolated sugar with the major pyranose tautomer (**1a**) dominating the spectrum. ^1^H NMR resonances of the minor furanose tautomer (**1b**) were mostly obscured by the major tautomer, but some signals were distinguished such as the anomeric protons at Glc*p* H-1′ (5.03 ppm, dd, 7.6 Hz, 3.9 Hz), Fru*f* H-3 (4.17 ppm, d, 3.8 Hz) and Fru*f* H-4 (4.15 ppm, d, 8.3 Hz). A table comparing the ^1^H NMR spectrum of the published and isolated trehalulose can be found in Table S1 of the supplementary material.

## Limitations

This fraction collection methodology should have applications to unknown sugars or other bioactive compounds separable by IC and that are not removed by the suppressor during neutralisation (i.e., not zwitterions or surfactants). Due to the time taken to concentrate the analyte of interest, time or temperature critical compounds would need further controls to be added to ensure stability during processing.

## Ethics statements

Not applicable.

## CRediT authorship contribution statement

**Hans S.A. Yates:** Conceptualization, Methodology, Formal analysis, Writing – original draft. **James F. Carter:** Supervision, Writing – review & editing. **Mary T. Fletcher:** Supervision, Writing – review & editing. **Viviene S. Santiago:** Writing – original draft, Formal analysis, Writing – review & editing. **Ondrea Thompson:** Resources, Visualization, Writing – original draft. **Natasha L. Hungerford:** Supervision, Writing – review & editing, Formal analysis.

## Declaration of competing interest

The authors declare that they have no known competing financial interests or personal relationships that could have appeared to influence the work reported in this paper.

## Data Availability

No data was used for the research described in the article.

## Appendix Supplementary materials

Image, application 1
