# Supplementary material for: Fraction collection of bioactive compounds from ion chromatography: No longer mission impossible
Source: MethodsX. 2025 Feb 21;14:103243. doi: 10.1016/j.mex.2025.103243 (PMC11919326; doi:10.1016/j.mex.2025.103243)
Supplement: Supplementary file 1 [file mmc1.docx]

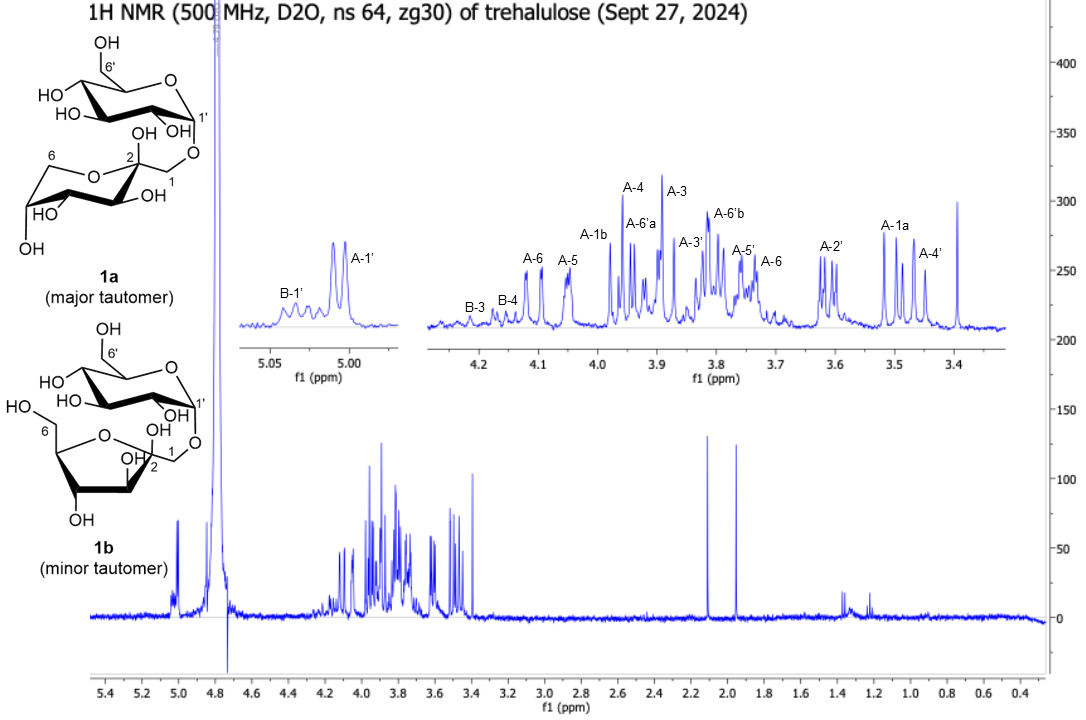


Fig. S1. ^1^H NMR (500 MHz, ns 64, D_2_O) spectrum of isolated trehalulose

Table S1. Comparison of ^1^H NMR data of isolated trehalulose with literature data for trehalulose.

| **Ring** | **Position** | **Experimental***  **δH, mult (*J in Hz*)** | **Literature****  **δH, mult (*J in Hz*)** |
| --- | --- | --- | --- |
| **1-O-α-D-glucopyranosyl-β-D-fructopyranose (1a)** | | | |
| Glc*p* | 1’ | 5.01 (*d, 3.7*) | 4.98 (*d, 3.7*) |
|  | 2’ | 3.61 (*dd, 9.8, 3.7*) | 3.58 (*dd, 9.9, 3.7*) |
|  | 3’ | 3.83 (m) | 3.79 (*dd, 9.5, 9.2*) |
|  | 4’ | 3.47 (*dd, 10.0, 9.2*) | 3.44 (*dd, 9.8, 9.5*) |
|  | 5’ | 3.76 (*td, 4.5, 2.2*) | 3.73 (*td, 9.8, 2.5*) |
|  | 6’ | a 3.91 (*dd, 12.2, 2.2*) | a 3.88 (*dd, 12.2, 2.5*) |
|  |  | b 3.81 (*dd, 12.6, 5.2*) | b 3.80 – 3.76 (*m*) |
| Fru*p* | 1 | a 3.51 (*d, 10.3*) | a 3.48 (*d, 10.3*) |
|  |  | b 3.97 (*br d, 10.3*) | b 3.94 (*br d, 10.3*) |
|  | 3 | 3.88 (*d, 10.0*) | 3.85 (*dd, 10.0, 0.9*) |
|  | 4 | 3.95 (*dd, 10.8, 3.5*) | 3.92 (*dd, 10.0, 2.0*) |
|  | 5 | 4.05 (*m*) | 4.02 (*dd, 2.0, 1.6*) |
|  | 6 | a 4.11 (*dd, 12.8, 1.3*) | a 4.08 (*ABdd, 12.8, 1.0*) |
|  |  | b 3.74 (*t, 2.0*) | b 3.72 (*ABdd, 12.8, 0.9*) |
| **1-O-α-glucosylpyranosyl-β-D-fructofuranose (1b)** | | | |
| Glc*p* | 1’ | 5.03 (*dd, 7.6, 3.9*) | 5.01 (*d, 3.7*) |
|  | 2’ | 3.61 | 3.59 |
|  | 3’ | 3.77 | 3.76 |
|  | 4’ | 3.47 | 3.44 |
|  | 5’ | 3.73 | 3.72 |
|  | 6’ | 3.90 | 3.89 |
| Fru*f* | 1 | 3.58 | 3.56 |
|  | 3 | 4.17 (*d, 3.8*) | 4.15 |
|  | 4 | 4.15 (*d, 8.3*) | 4.13 |
|  | 5 | 3.91 | 3.88 |
|  | 6 | 3.85 | 3.81 |

*^1^H (600 MHz, 64 scans) recorded in D_2_O

**^1^H (600 MHz, 32 scans) recorded in D_2_O, data from Fletcher *et al.* (2020)
